# A machine learning model to predict the risk factors causing feelings of burnout and emotional exhaustion amongst nursing staff in South Africa

**DOI:** 10.1186/s12913-024-12184-5

**Published:** 2024-12-31

**Authors:** Maria Magdalena Van Zyl-Cillié, Jacoba H. Bührmann, Alwiena J. Blignaut, Derya Demirtas, Siedine K. Coetzee

**Affiliations:** 1 Faculty of Engineering, North-West University, 11 Hoffman Street, Potchefstroom, South Africa; 2 Faculty of Behavioural, Management and Social Sciences, University of Twente, 5 Drienerlolaan, 7522 NB Enschede, The Netherlands; 3 NuMIQ Research Focus Area, School of Nursing Science, North-West University, 11 Hoffman Street, Potchefstroom, South Africa

**Keywords:** Supervised machine learning model, Nurse burnout, Emotional exhaustion, Maslach Burnout Inventory

## Abstract

**Background:**

The demand for quality healthcare is rising worldwide, and nurses in South Africa are under pressure to provide care with limited resources. This demanding work environment leads to burnout and exhaustion among nurses. Understanding the specific factors leading to these issues is critical for adequately supporting nurses and informing policymakers. Currently, little is known about the unique factors associated with burnout and emotional exhaustion among nurses in South Africa. Furthermore, whether these factors can be predicted using demographic data alone is unclear. Machine learning has recently been proven to solve complex problems and accurately predict outcomes in medical settings. In this study, supervised machine learning models were developed to identify the factors that most strongly predict nurses reporting feelings of burnout and experiencing emotional exhaustion.

**Methods:**

The PyCaret 3.3 package was used to develop classification machine learning models on 1165 collected survey responses from nurses across South Africa in medical-surgical units. The models were evaluated on their accuracy score, Area Under the Curve (AUC) score and confusion matrix performance. Additionally, the accuracy score of models using demographic data alone was compared to the full survey data models. The features with the highest predictive power were extracted from both the full survey data and demographic data models for comparison. Descriptive statistical analysis was used to analyse survey data according to the highest predictive factors.

**Results:**

The gradient booster classifier (GBC) model had the highest accuracy score for predicting both self-reported feelings of burnout (75.8%) and emotional exhaustion (76.8%) from full survey data. For demographic data alone, the accuracy score was 60.4% and 68.5%, respectively, for predicting self-reported feelings of burnout and emotional exhaustion. Fatigue was the factor with the highest predictive power for self-reported feelings of burnout and emotional exhaustion. Nursing staff’s confidence in management was the second highest predictor for feelings of burnout whereas management who listens to employees was the second highest predictor for emotional exhaustion.

**Conclusions:**

Supervised machine learning models can accurately predict self-reported feelings of burnout or emotional exhaustion among nurses in South Africa from full survey data but not from demographic data alone. The models identified fatigue rating, confidence in management and management who listens to employees as the most important factors to address to prevent these issues among nurses in South Africa.

## Background

With an increasing demand for healthcare services, nursing staff are pressured to provide quality care with limited human and material resources. Dubale et al. [1] explain that the demanding work environment of nurses has undesirable consequences such as burnout and physical and emotional exhaustion. In fact, the prevalence of burnout and other mental morbidities is more significant in the healthcare workforce than among workers in other settings [2–4]. Burnout is a chronic response to stress in the workplace, characterized by a physical, mental and emotional state of exhaustion that reduces the nurse’s sense of personal and professional fulfillment [5].

Determining factors that cause burnout amongst nursing staff can be done through statistical analysis of survey responses to questionnaires. Researchers such as Grochowska et al. [6] and Kowalski et al. [7] have done so effectively by analysing nurses’ responses to their own questionnaires and the standardized Maslach Burnout Inventory tool. Although their findings provide insight into the factors that cause burnout amongst nursing staff, such analysis is often reactive and after the fact. Specifically, their research focuses on detecting burnout and the factors that lead to burnout from survey responses rather than predicting factors that cause burnout [7]. Subsequently, Carvalho Manhães Leite and Wooldridge [8] call for novel methods to predict the factors that lead to burnout amongst nursing staff instead of detecting the factors retrospectively.

To this end, Machine Learning (ML) can be used to predict burnout and the factors with the highest probability of causing burnout. Bzdok and Krzywinski [9] state that statistical methods draw population inferences from samples, whereas ML finds generalisable patterns that can be used for prediction. Recent studies have also shown that ML algorithms are able to handle complex data and have outperformed traditional statistical models in the prediction of several variables [10–13].

Specifically, supervised ML is a branch of computer engineering where computational methods use experience in the form of historical data, with provided outcomes, to train a computer model to make accurate predictions [14]. It has proven to be an effective technique to handle complex problems in many fields, including healthcare. Char et al. [15] agree that incorporating ML into clinical medicine studies holds promise for substantially improving healthcare delivery. Alzu’bi et al., [16], for example, presented a model to predict and reduce absenteeism of nurses. Grzadzielewska [17] emphasized the effectiveness of utilising ML to predict burnout and Havaei et al. [18] successfully employed ML modelling to determine the most crucial workplace factors that affect the mental health of nurses.

Furthermore, Adapa et al. [19] demonstrated that ML can identify the factors with the highest predictive power to predict burnout among healthcare workers. Their study, however, was limited to a small sample from one medical centre and they conclude that further studies are necessary to explore ML’s potential in identifying targeted interventions for preventing healthcare worker burnout. Therefore, predicting burnout from survey data using a tool like machine learning (ML) would be beneficial.

Several antecedents to burnout amongst nurses have been identified in the literature; the most prominent is the nurse's practice environment and personal factors, such as incompatibility between work and private life. These can also include socio-demographic characteristics, organizational and healthcare system issues, and patient outcomes [20–22]. Understanding the specific factors that lead to burnout and emotional exhaustion of nurses is critical to primarily providing the appropriate support to nurses [23] and subsequently informing the development of mitigating policies [20, 24].

Even though a plethora of international literature on nurse burnout and emotional exhaustion is available, studies on burnout among healthcare workers in sub-Saharan Africa are limited. Although some researchers such as Owuor et al. [25], Moyo et al. [26] and Van der Doef [27] have focused their studies on burnout of nurses in this region, Brückner et al. [28] contend that very little is known about the unique factors and risks associated with burnout and emotional exhaustion of healthcare workers in Sub-Saharan Africa. A systematic review by Woo et al. [29] confirms that Sub-Saharan Africa has the highest incidence of burnout among nurses globally. We note that most studies within this context are descriptive in nature, providing factors most associated with burnout [30–32] and do not use ML as a method to predict these factors. This limits the recommendations posed to after-the-fact treatment and containment rather than providing solutions for early detection and prevention.

While Maslach’s multidimensional theory conceptualises burnout in terms of three components, namely emotional exhaustion, depersonalisation and reduced personal accomplishment [28], depersonalisation is seen as the interpersonal dimension of burnout,while personal accomplishment is seen as the self-evaluation dimension of burnout [33]. Emotional exhaustion, on the other hand, is seen as the basic individual stress dimension of burnout, having its major source in work overload or personal conflict at work. Emotional exhaustion is also an orthodox stress variable [33] and is therefore more predictive of negative health outcomes than the other two dimensions. Focusing on emotional exhaustion, Maslach [33] states that it is the most widely reported of the three aspects of burnout, being the central quality and the most obvious manifestation of this complex syndrome. Additionally, Li-Sauerwine [34] found that the single question “I feel burned out from my work” in the 9-item Maslach Burnout Inventory tool has proven to be a good predictor of burnout and can be used as a rapid screening tool for burnout. We contend that this question indicates whether respondents experience feelings of burnout.

Our research aims to highlight the distinct factors that can strongly predict feelings of burnout and emotional exhaustion among nurses in South Africa. To this end, this research has three objectives of which the first is to determine which factors most prominently predict that nursing staff in South Africa will indicate that they have feelings of burnout once a week or more frequently. The second objective is to identify factors that can predict high emotional exhaustion within the group, as measured by the Maslach Burnout Inventory instrument. The third objective of the study is to determine the accuracy of the predictions, and the prominent factors found when only using demographic data versus incorporating survey responses.

## Methodology

This study employed a cross-sectional descriptive design to utilise survey data combined with an ML method [35]. The study and reporting thereof followed the standard STROBE checklist typically used in cross-sectional studies [36]. The method to construct the supervised ML model was followed according to the suggestion by Tsai et al. [37]. The outcomes of the ML model were further validated by survey data analysis.

The remainder of this section describes the nationwide cross-sectional survey data that was used, as well as the ethical considerations. Next, an explanation of the preparation conducted on the survey data for ML modelling is provided, including a description of the feature set and target variables in the data extraction. This is followed by a description of the ML model development used to determine the most prominent features that predict the target variables. Finally, the model evaluation method is explained.

### Data collection

The data for this research was collected from a cross-sectional survey [35, 38–42] where multi-phase sampling was used to recruit all categories of nursing staff in the private and public hospitals of South Africa [43]. The cross-sectional survey aimed to assess nurse outcomes, quality of care, and patient safety in 143 hospitals across the 9 provinces of South Africa during the COVID-19 pandemic. Data collection started in April 2021 and concluded in June 2022 via online surveys (private hospitals) and paper-based surveys (public hospitals) as requested by the different healthcare sectors. All in-patient units were included. A total population sample of registered and enrolled nurses and enrolled nursing assistants from sampled units was included. This study only used medical-surgical unit data, to ensure homogeneity of the sample. The survey constituents are presented in Table 1.

**Table 1:** Description of instruments used in the survey

Construct measured	Instrument	Subscales or items	Cronbach alpha in current study
**Demographics**	Demographic Data	Nine questions: 1. What is your gender? 2. What is your current employment status? 3. What is your nurse category? 4. Do you have a bachelor’s degree in nursing? a. If yes, at what level is your speciality training? b. If yes, in which discipline is your speciality training? 5. What is your age? 6. How many years have you worked as a nurse? 7. How many years have you worked in your current hospital? 8. Speciality area of your current unit?	Single items – not relevant
**Career and job satisfaction**	RN4CAST [44]	Career satisfaction: 1 question ranging from 1 (Very dissatisfied) to 4 (Very satisfied)	Single item – not relevant
		Job satisfaction: 11 questions ranging from 1 (Very dissatisfied) to 4 (Very satisfied)	
**Job and career turnover intent**	RN4CAST [44]	Job turnover intent: 1 question with options for 1 (yes) or 2 (no)	Single items – not relevant
		Career turnover intent: 1 question with options for 1 (Nursing in another hospital), 2 (Nursing not in a hospital) or 3 (Non-nursing career)	
**Practice environment**	PES-NWI [45]	Nurse participation in hospital affairs: 9 questions Nursing foundations for quality of care: 11 questions Nurse manager ability, leadership and support of nurses: 6 questions Staffing and resource adequacy: 4 questions Collegial nurse-physician relationships: 6 questions Overall: 35 questions ranging from 1 (Strongly disagree) to 4 (Strongly agree)	Nurse participation in hospital affairs: 0.88 Nursing foundations for quality of care: 0.84 Nurse manager ability, leadership and support of nurses: 0.84 Staffing and resource adequacy: 0.75 Collegial nurse-physician relationships: 0.89
**Overall practice environment**	RN4CAST [44]	One question ranging from 1 (Poor) to 4 (Excellent)	Single item – not relevant
**Resources**	RN4CAST [44]	Four questions ranging from 1 (Frequently) to 4 (Never)	Single items – not relevant
**Workplace violence**	Workplace Relationships Negative Acts Questionnaire [46]	Physically intimidating bullying: 3 questions Person-related bullying: 12 questions Questions ranging from 1 (Never) to 5 (Daily)	Physically intimidating bullying: 0.83 Person-related bullying: 0.96
**COVID-19**	Item developed for present study	One question ranging from 1 (Never) to 4 (Routinely)	Single item – not relevant
**Death and dying**	Item developed for present study	One question ranging from 1 (Never) to 4 (Routinely)	Single item – not relevant
**Quality of care**	RN4CAST [44]	Four questions ranging from 1 (Very confident / Excellent / Definitely yes) to 4 (Not at all confident / Poor / Definitely no)	Single items – not relevant
**Patient safety**	RN4CAST [44]	Patient safety: 1 question ranging from 1 (Excellent) to 5 (Failing) Patient safety culture: 8 questions ranging from 1 (Strongly agree) to 5 (Strongly disagree)	Single items – not relevant
**Adverse events**	RN4CAST [44]	Nine questions ranging from 1 (Never) to 5 (Daily)	Single items – not relevant
**Health and wellbeing**	Items developed for present study	Five questions with options 1 (Yes), 2 (No) or 3 (Unknown)	Single items – not relevant
**Absenteeism**	Items developed for present study	One question on incidence of being absent measured in days, one question with options for 1 (Physical illness), 2 (Injury), 3 (Family responsibility) or 4 (Mental health day)	Single items – not relevant
**Burnout**	Maslach Burnout Inventory Instrument [28]	Emotional exhaustion: 9 questions ranging from 1 (Never) to 7 (Every day)	Emotional exhaustion: 0.89
**Compassion practice**	Compassion Practice Instrument [35]	Compassion satisfaction: 5 questions Disengaged: 6 questions Impotent: 4 questions Unfulfilled: 5 questions Overall: 20 questions ranging from 1 (Never) to 6 (Always)	Compassion satisfaction:0.84 Disengaged: 0.75 Impotent: 0.84 Unfulfilled: 0.64
**Global health**	Global Health Short Form [47]	Physical health: 4 questions Mental health: 6 questions Overall: 10 questions ranging from 1 (None / Excellent) to 5 (Very severe / Poor)	Physical health: 0.73 Mental health: 0.79

### Ethical considerations

The Health Research Ethics Committee of the North-West University provided ethical approval for data collection on the health and well-being nursing staff in South Africa [35, 38–42], which was carried out in accordance with the Declaration of Helsinki [48]. The committee's ethics number is NWU-00033–19-A1. The health authorities and specific public health facilities in each of the nine provinces of South Africa granted permission to carry out the study. A comprehensive information sheet and an oral explanation of the study were provided to each participant. It was also explained that their participation in the research was voluntary and private. Each participant gave their informed consent in writing before beginning the cross-sectional survey.

### Data preparation and imputation

The survey data was used as the input to train various supervised ML models to predict the factors causing feelings of burnout and emotional exhaustion in nurses. In supervised ML models, an algorithm is trained on known inputs (features) and their corresponding outputs (target variables). The trained algorithm then aims to predict the correct outcomes given similar inputs [49]. Data preparation and imputation were done as follows.

#### Data preparation

The dataset included 1165 survey responses, with each participant's responses in individual rows and each question answer in columns (refer to Table 5 of [35]). One of these columns contained the value to be predicted, known as the target variable. The rest were input features.

To derive the binary target variables in this research, we used the responses to the 9-item Maslach Burnout Inventory instrument [28]. For the purpose of this research, we use the acronym MBI to refer to the Maslach Burnout Inventory indicators that were used as target variables as explained in detail below.

Since the first objective of this research is to determine the factors that will most prominently predict that nursing staff in South Africa will indicate that they have feelings of burnout once a week or more, the first target variable was derived from question 5 of the 9-item Maslach Burnout Inventory instrument item (*“*I feel burned out from my work*”*), with respondents indicating that they felt burned out once a week or more classified positive, and respondents who indicated feelings of burnout less than once a week as negative. We named this target variable MBI-Q5.

For the second target variable, our focus was on predicting emotional exhaustion among respondents. In accordance with the guidelines of Maslach et al. [50], respondents with a score of 27 or more on the nine Maslach Burnout Inventory survey questions were classified as experiencing high emotional exhaustion. To address the second objective of our research, we created a second target variable (referred to as MBI) that could be used to identify the factors that can predict high emotional exhaustion within the group, as measured by the 9-item Maslach Burnout Inventory instrument. Positive cases of the MBI target variable in this research, therefore, refer to respondents that achieved a score of 27 or higher on the 9-item Maslach Burnout Inventory instrument survey questions. Likert-scale and category responses were coded as categorical variables, while continuous data like age and number of years in the nursing profession, were coded as numerical variables.

#### Data imputation

Literature on practical guidelines for handling missing data [51] suggests that it is preferable to use imputation techniques to handle missing data rather than to ignore or delete the data. However, Jakobsen et al. [52] argue that inferences drawn from a dataset with more than 40% missing values are regarded as hypothetical. They also suggest that missing values can be ignored when the total number of deleted values is less than 5% of the dataset.

In this research, four of the initial 138 features (2% of features) were deleted based on more than 40% of the values being missing. The four deleted features were the Body Mass Index of each respondent, their response on whether they were part of a team, the number of people in their team and where respondents would work if they intended to leave their job. For features with less than 40% missing values, a simple imputation strategy, as described by Jakobsen et al. [52], was followed by replacing missing numerical values with the mean of the feature. In accordance with the recommendation of Makaba and Dogu [53] missing categorical values were imputed with the mode of the feature. Since features that highly correlate with the target variable have a high impact on the accuracy of classification in ML models [54].

Eight features from the Maslach Burnout Inventory instrument that showed a high statistical correlation with the target variable were removed to prevent overfitting of the model. This resulted in a dataset with 126 input features, of which the categorical features were split into binary classification features as required by the ML model.

As the self-reported burnout target variable (MBI_Q5) is the response on how often respondents feel burned out from their work, the six respondents who did not answer this question in the survey were deleted from the dataset. This deletion was done after it was confirmed that there were no specific similarities from any of the responses to the other survey questions of these participants. Since the MBI target variable is calculated from the Maslach Burnout Inventory survey questions (and positive cases of high emotional exhaustion are respondents who score 27 or more on the 9 questions), these respondents were also removed when classifying respondents for emotional exhaustion. The number of respondents that remained in the analysis was, therefore, 1159.

To predict the two target variables, the dataset was duplicated: Subset 1 with all 126 features predicting MBIQ5, and Subset 2 predicting high emotional exhaustion (represented by a 9-item MBI score of 27 or more). Both subsets contained the same 1159 responses, and the only difference between the subsets was the target variables included to train the ML models. The data was split into training/testing and unseen datasets using a 75%−25% ratio. This implied that 75% of the data was used for training and testing whereas 25% was kept separate to evaluate the final prediction model(s). Of the 75% training and testing data, 70% was used to train models and 30% to test the models so that hyperparameter tuning could be performed.

### Model development and evaluation

Anaconda Navigator was used as the graphical user interface and JupyterLab 4.0.11 as the interactive development environment to execute the Python 3.11.7 code of the supervised classification ML models. Similar to the method suggested by Tsai et al. [37], the PyCaret 3.3 package [55] was used to automate the ML workflow. This allowed for the simultaneous training and evaluation of 18 types of classification models. In addition, the PyCaret 3.3 package was employed to perform the data imputation strategy as explained in the "Data imputation" section. The built-in functions of PyCaret 3.3 were then deployed to split the training and test data randomly. Subsequently, classification ML models were trained with the training set and evaluated with the test set, using ten fitting folds. By inspecting Subset 1 and Subset 2, we found that there was a balanced representation of both binary values for each target variable. This led us to choose the accuracy metric to identify which ML models would be most suitable and have the highest prediction power. Because the data was balanced, we did not have to consider other metrics like recall and precision that come into play in unbalanced prediction classification models [56].

The next step included the hyperparameter tuning of the top models that had the potential to be the best model; this is often referred to as hyper-tuning and differs per model, as each model has its own set of hyperparameters. The hyperparameter tuning of the ML models was performed using the tuning function of the PyCaret 3.3 package [55]. This function uses a random grid search (RGS) to tune the various hyperparameters related to each model. RGS evaluates model performance according to predefined parameter combinations to determine the best combination [55].

Once the best ML model and corresponding hyperparameters for both subsets and their corresponding target variables were determined, the models were used to evaluate the prediction performance of the test dataset. This evaluation was done by means of a confusion matrix and the receiver operating characteristic (ROC)-curve [14].

A confusion matrix plots the actual values of the target variables compared to the predicted outcomes, classifying them as True Positive (TP), False Positive (FP), True Negative (TN), and False Negatives (FN) [38]. The ROC curve is a plot of the FP rate (x-axis) versus the TP rate (y-axis) [30]. The FP and TP rates were expressed as a value between 0 and 1. The ideal is to have the FP and FN rates as small as possible and the TP and TN rates as close to one as possible. To summarize the ROC curve, a single value known as the Area Under the Curve (AUC) is represented by a value between 0 and 1. The most accurate AUC value of 1 will indicate that the ML model predicts only TP and no FP values [30] and can make perfect predictions. This implies that the AUC value can be used to indicate the overall ranking performance of a classifier [14, 56].

The final phase of the model development was to finalise the models and evaluate the prediction strength of the tuned models for each target variable using the unseen data. Consequently, the ML models that were trained and tested in the previous step were evaluated on the reserved 25% of the original data that was left as unseen data. The evaluation was done using another confusion matrix, this time for the unseen data and by assessing each ML model's final accuracy score.

The features with the highest predicting power were extracted and ranked. Since each of these features corresponds to a question in the survey data, the factors that most strongly predicted whether a nurse would respond to the question “I feel burned out from my work” as once a week or more (MBI_Q5) or suffer from high emotional exhaustion (MBI) could be determined.

Lastly, the exercise was repeated for both target variables (MBI_Q5 and MBI) using only demographic data to determine how accurate predictions can be made without full survey knowledge. To achieve this objective, two additional ML models were trained and tested as described in the process above. The subsets for these additional models consisted of only demographic information and the MBI_Q5 target variable for the first model, and demographic information and the high emotional exhaustion (MBI) target variable for the second model. There were 11 input features in each demographic dataset. As before, the two demographic models were also evaluated on unseen data to obtain an accuracy score. The features of the two demographic models with the strongest predictive power were compared to those of the full survey data ML models. The accuracy scores and feature comparisons were used to determine whether feelings of burnout once a week or more (MBI_Q5) and high emotional exhaustion (MBI) could accurately be predicted using only demographic data.

## Results

In this section, we elaborate on the results of our research by first presenting the five models that most accurately predicted the target variables of subsets 1 and 2 and subsequently present the model for each subset that performed the best in terms of accurate predictions after refinement (known as hyperparameter tuning) was completed. We then show which features the models on full survey data indicated as having the highest probability of predicting that nurses in South Africa’s response to the question “I feel burned out from my work” will be “once a week” or more frequently (Subset 1) and which features most strongly predicted that nurses in South Africa will experience high emotional exhaustion (Subset 2). By presenting these results, we address the first two objectives of the research.

To address the third objective of this research, we also present the accuracy score of ML models for the two target variables based only on demographic survey data. Lastly, we confirm the outcomes from our models by completing descriptive statistical analysis of the survey data to determine whether the survey responses confirm the outcomes of the ML models.

In the "Model Evaluation" section the predictive accuracy scores of the top five models of the evaluated 18 ML models used by the PyCaret 3.3 package [55] are presented. All the top five models for both target variables (MBI_Q5 and MBI) were selected for hyperparameter tuning. This was done to ensure the most accurate ML models after finetuning were selected for both subsets 1 and 2. Next, the confusion matrixes, ROC-curves, and AUC scores of the test data of the finetuned models that resulted in the highest accuracy predictions of each of the target variables are presented. The results when testing the models on the unseen data are presented next, including the confusion matrixes and accuracy scores for each respective subset. Thereafter, the accuracy results of the ML models that were trained, tested, and evaluated using demographic data only, are presented. The "Feature predictions" section of the results section focuses on the features that the ML models listed as having the strongest predicting power for the MBI_Q5 and high emotional exhaustion target variables, respectively. The features are compared with those of the full survey data results. The study results are concluded in the "Descriptive Statistics" section by performing descriptive statistics on the strongest predicting features to confirm the validity of the ML models further.

### Model Evaluation

The top five performing models before hyperparameter tuning for Subset 1 and Subset 2 are shown in Table 2.

**Table 2:** Top five performing supervised ML models based on test data

Subset 1 (Target variable = MBI_Q5)		Subset 2 (Target variable = MBI)	
Model	Accuracy %	Model	Accuracy %
Gradient Booster Classifier	76.98%	Gradient Booster Classifier	76.8%
Extra Trees Classifier	75.83%	Extra Trees Classifier	76.47%
Light Gradient Boosting Machine	75.17%	Random Forest Classifier	75.33%
Naive Bayes	72.84%	Light Grading Boosting Machine	75.15%
Random Forest Classifier	72.69%	Ada Boost Classifier	73.68%

The gradient booster classifier (GBC) model performed the best to predict both MBI_Q5 and high emotional exhaustion (MBI). A GBC model is built in stages where a simple model, such as a decision tree, is used as a base to predict the target variable. Thereafter, the loss function (residual errors) of these predictors is optimized in stages by fitting a new model on the residuals before it is generalised [57].

**Fig. 1:**
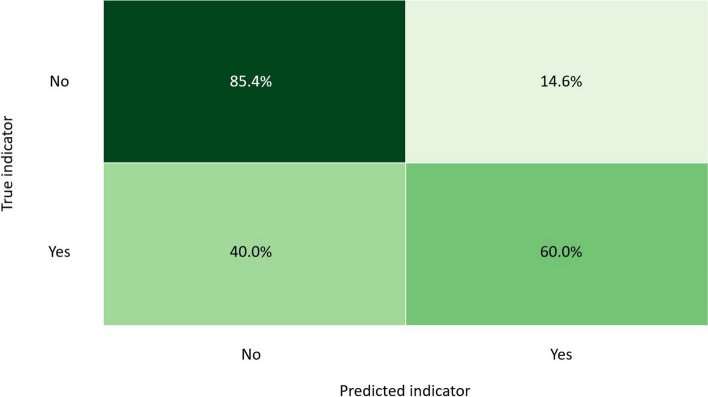
GBC model confusion matrix of test data Subset 1 (MBI_Q5)

For both subsets, the top five models underwent hyperparameter tuning. The tuned models indicated that the original trained GBC models remained the best model for both subsets. The accuracy score of the test data for the GBC model predicting MBI_Q5 was 76.98% whereas the accuracy score of the GBC model to predict high emotional exhaustion was 76.8%. The confusion matrix of the test sets for MBI_Q5 is shown in Fig. 1.

The confusion matrix for MBI_Q5 indicates that for all the positive = “Yes” cases of MBI_Q5, the model predicted 60% correctly on the test data. In the cases where MBI_Q5 was negative = “No”, the majority of the predictions (85.4%) on the test data were done correctly. We therefore conclude that the sensitivity of the model on the test data is 60% and the specificity 85.4%.

**Fig. 2:**
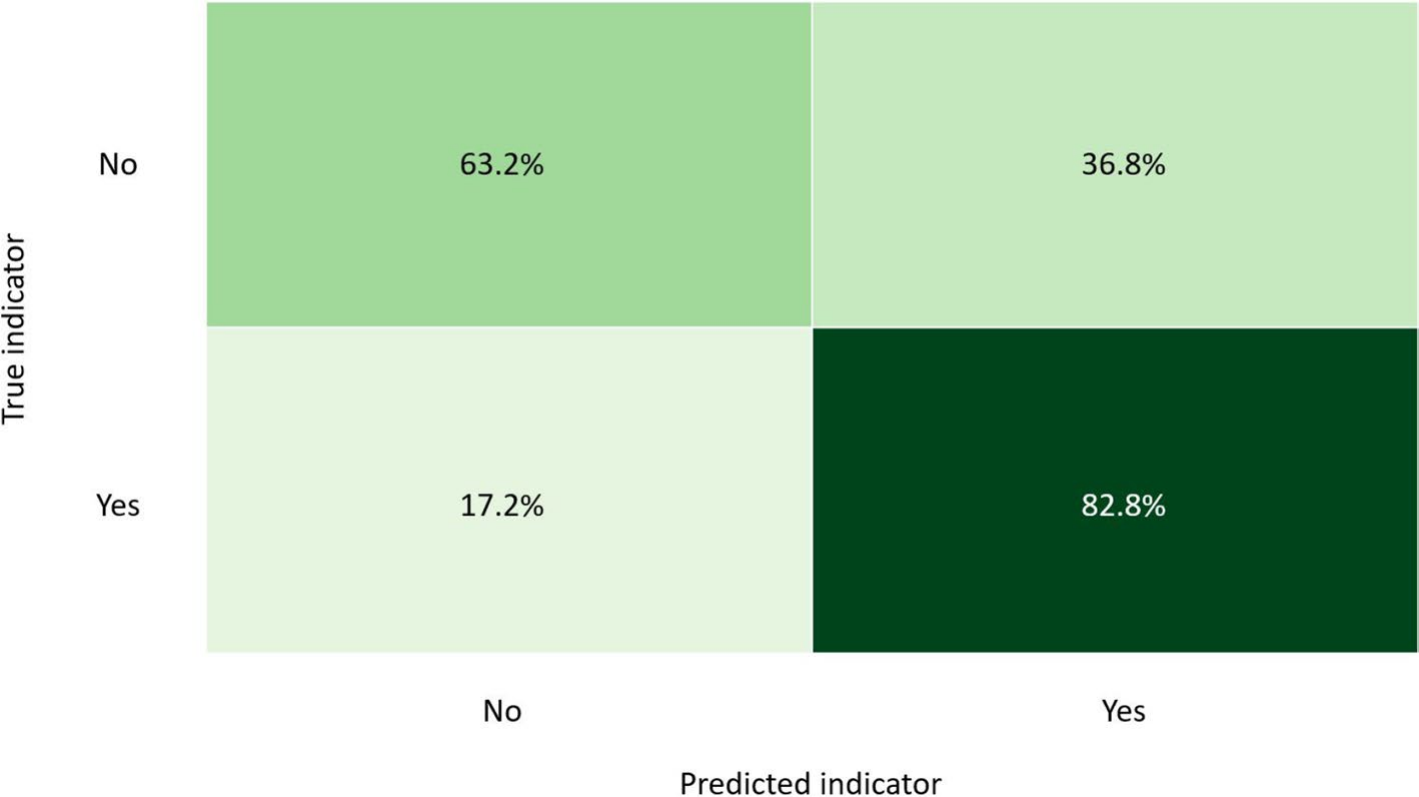
GBC model confusion matrix of test data Subset 2 (MBI)

Figure 2 depicts the confusion matrix of the test set for the best-found GBC model for the high emotional exhaustion instances (MBI).

The predictions by the GBC model in Fig. 2 show that 89.3% of the high emotional exhaustion instances could be predicted correctly for all the positive = “Yes” cases (sensitivity = 89.3%). However, in cases where high emotional exhaustion was not present, the model predicted only 57.6% of the negative high emotional exhaustion predictions correctly on the test data. The specificity was, therefore, 57.6%.

**Fig. 3:**
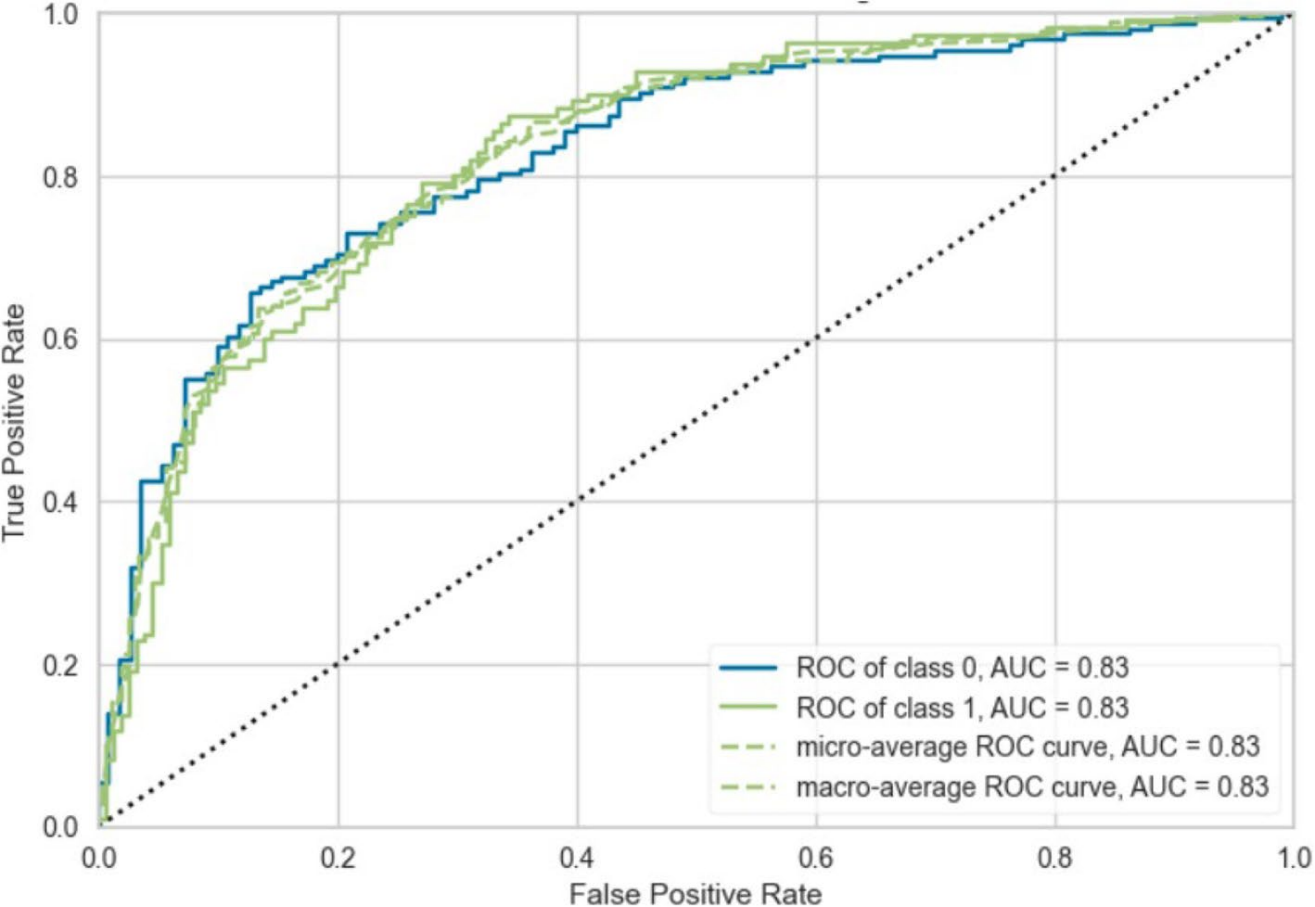
ROC-curve of Subset 1 (MBI_Q5)

**Fig. 4:**
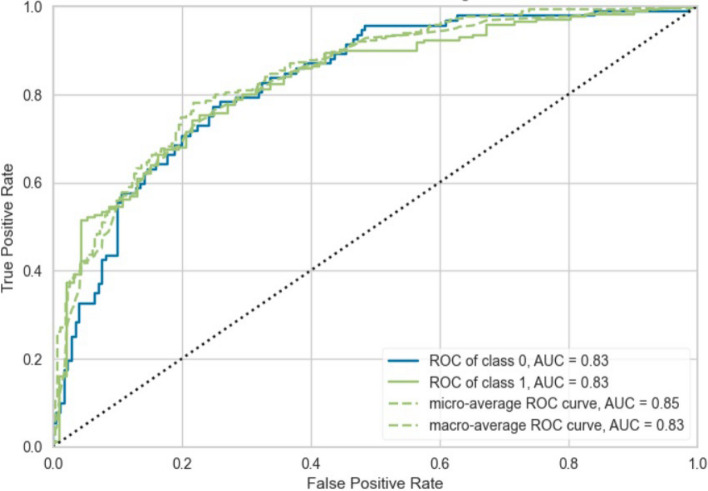
ROC-curve of Subset 2 (MBI)

The ROC-curves were also inspected to determine the TP versus FP rates. Figure 3 is the ROC-curve of subset 1 (MBI_Q5), and Fig. 4 is the ROC-curve of Subset 2 (MBI).

The ROC-curve of subset 1 in Fig. 3 indicates that there is a strong tendency of the first GBC model to predict TP values for the MBI_Q5 target variable rather than FP values. This trend can also be seen in Fig. 4, with an even stronger rate of TP value prediction for the high emotional exhaustion target variable. (MBI). The AUC score for the MBI-Q5 model was 0.83, and for the MBI model, 0.85. In their case study on burnout of healthcare practitioners using a supervised ML model, Adapa et al. [19] conclude that an ML model with an AUC of 0.81 can accurately predict burnout in healthcare professionals. The models in this study performed better on the test data of both subsets in terms of AUC scores.

**Fig. 5:**
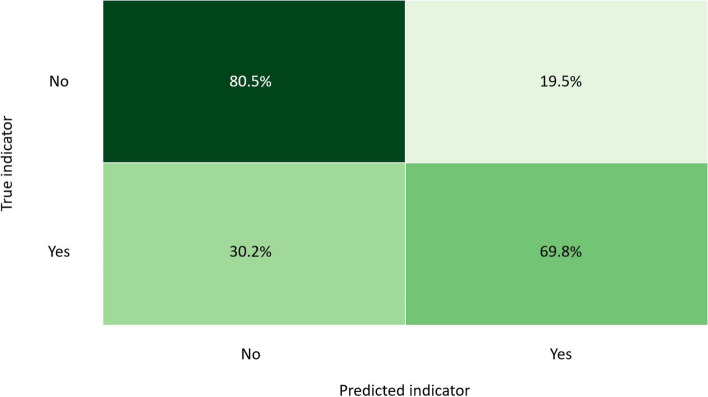
GBC model confusion matrix of unseen data Subset 1 (MBI_Q5)

The final step in evaluating the models was to make predictions using 25% of the original dataset that was reserved as unseen data. The accuracy of the GBC model to correctly predict MBI_Q5 was 75.8%. Figure 5 is the confusion matrix of the GBC model on unseen data for the prediction of MBI_Q5.

**Fig. 6:**
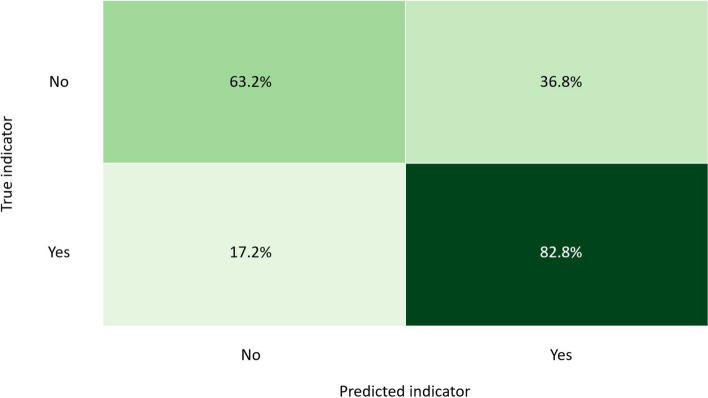
GBC model confusion matrix of unseen data Subset 2 (MBI)

The model correctly predicted cases of MBI_Q5 in 69.8% of TP values but performed better by classifying TN values of MBI_Q5 correctly in 80.5% of the cases. The model, therefore, demonstrates a sensitivity of 69.5% and specificity of 80.5% on unseen data The accuracy score of the GBC model on the unseen high emotional exhaustion data was 76.8%. The confusion matrix for this model on the unseen data is in Fig. 6.

In predictions on unseen data, TP values of high emotional exhaustion were correctly indicated in 82.8% of the instances (sensitivity = 82.8%). Although there is a slight improvement in terms of correctly predicting TN cases of high emotional exhaustion, the model still classified 36.8% of TN values of high emotional exhaustion as positive cases. Furthermore, because the overall accuracy score of the model is 76.8%, the ML model can accurately predict cases of high emotional exhaustion.

Overfitting, where the ML algorithm mimics the training data instead of learning from it, is often a risk when training an ML model [14]. If the metrics, such as model accuracy, differ significantly between the testing predictor strength and the unseen data, it indicates that the model was overfitted. However, it was found that the model for MBI_Q5 performs similarl on the test data and unseen data in terms of accuracy (76.98% on test data versus 75.8% on unseen data). The model to predict high emotional exhaustion also did not indicate an instance of overfitting since the accuracy score on the test data was 76.8%, which is the same as the 76.8% accuracy score on the unseen data. Therefore, comparisons between the test and unseen data metrics indicated that the results were close enough to assume overfitting was not an issue.

Lastly, the results from training and testing ML models to predict MBI_Q5 based only on demographic data have shown much lower accuracy scores. When demographic data was used to predict MBI_Q5, the accuracy score was 60.4% and in the case of high emotional exhaustion it was 68.5%.

The results of this study indicate that both the occurrence of respondents feeling burned out from their work once a week or more (MBI_Q5) and high emotional exhaustion can more accurately be modelled and predicted by including the full survey features rather than only using features related to demographic information. Furthermore, demographic features were not listed as having the highest predictive power of the target variable outcomes in either of the models. We do, however, note that in cases where full survey data is unavailable, the demographic models have some extent of predictive power, especially in the case of predicting emotional exhaustion, where the accuracy score achieved is close to 70%.

### Feature predictions

Since 138 features can potentially predict the two target variables (MBI_Q5 and MBI), a feature with a higher predictive power than one 138th (or 0.7%) can be seen as having a stronger predictive power than the other features. In our approach to determine the factors with the highest predictive power for the two target variables, we take a conservative approach and list the top 12 features with a predictive power of 1.5% or more for each model of the full survey data.

Table 3 lists features from the full survey data that have the strongest predictive power that nurses will indicate they have feelings of burnout once a week or more (MBI_Q5).

**Table 3:** Highest predictive features of MBI_Q5 from full survey data

Feature	Feature description	Predictive power
F173	Rated Fatigue	8.03%
F135	Confidence in Management	4.67%
F27	Overall Job Satisfaction	4.11%
F85	Management listens to employees	3.77%
F169	Pain rating	3.51%
F134	Overall Patient Safety	2.88%
F172	Experience Emotional Problems	2.30%
F70	Enough registered nurses	2.30%
F137	Recommend place of work to receive healthcare services	2.24%
F161	Amount of workday spent on non-nursing tasks	2.07%
F29	Satisfaction with work-schedule	1.82%
F84	Extent to which nursing manager backs nursing staff	1.54%

With 11 demographic data features that can possibly predict MBI Q5, any feature with a predictive power of more than one 11th (or 9.09%) has a higher power of prediction than the other features. Table 4 shows the top five demographic features with the highest predictive power for MBI Q5.

**Table 4:** Highest predictive features of MBI_Q5 from demographic data

Feature	Feature description	Predictive Power
F170	Absenteeism	30.0%
F23	Number of years worked in hospital	24.0%
F15	Number of years worked as a nurse	16.0%
F12	Age	14.0%
F21	Speciality discipline	4.0%

It should, however, be emphasised that the predictive power indicates the impact the feature had on the training of the model that resulted in the reported accuracy score and should be contextualised accordingly. For example, in the demographic model, the predictive power of “Absenteeism” is 30% for MBI_Q5, whereas the predictive power of “Rated fatigue” to predict MBI_Q5 in the full survey data is 8%. This should not be interpreted as “Absenteeism” having a higher predictive power than “Rated fatigue” in general since the evaluation metrics, such as accuracy for the models, should be considered. Additionally, none of the demographic features were ranked as having high predictive power in the full survey data models. The feature that had the strongest predictive power to predict both MBI_Q5 and high emotional exhaustion (MBI) was rated fatigue. Confidence in management and overall job satisfaction ranked second and third highest, respectively, to predict MBI_Q5. The features from the full survey data that have the highest predictive power for high emotional exhaustion are listed in Table 5, where management that listens to employees and confidence in management are the second and third strongest predictors of emotional exhaustion.

**Table 5:** Highest predictive features of high emotional exhaustion (MBI) from full survey data

Feature	Feature description	Predictive power
F173	Rated Fatigue	5.26%
F85	Management listens to employees	2.91%
F135	Confidence in Management	2.71%
F27	Overall Job Satisfaction	2.37%
F70	Enough registered nurses	2.27%
F172	Experience Emotional Problems	2.02%
F137	Recommend place of work to receive healthcare services	1.81%
F162	Incomplete nursing care	1.77%
F169	Pain rating	1.68%
F161	Amount of workday spent on non-nursing tasks	1.57%
F73	Enough staff on hand to get the work done	1.54%
F23	Number of years worked in current hospital	1.51%

Similar to our approach on the demographic features that predict MBI_Q5, we list the top five demographic features with the highest predictive power to predict MBI in Table 6.

**Table 6:** Highest predictive features of high emotional exhaustion (MBI) from demographic data

Feature	Feature description	Predictive Power
F23	Number of years worked in hospital	19.0%
F12	Age	19.0%
F15	Number of years worked as a nurse	17.0%
F170	Absenteeism	15.0%
F17	Speciality area of current unit	6.0%

Interestingly enough, the demographic features that predict MBI_Q5 and high emotional exhaustion the strongest were not the same as the highest predictive features of the full survey data. In the case of MBI_Q5, absenteeism has the highest predictive power for the demographic data model. The number of years worked in a hospital was the feature from the demographic data that had the strongest predictive power of high emotional exhaustion. Our results confirm that it is indeed preferable to use survey data for the prediction of nurses to indicate that they will feel burned out from their work once a week or more and high emotional exhaustion if available.

We note that the top four features of the full survey data are the same for both predicting MBI_Q5 and MBI although their ranking differ. The same is seen on the ML models based on demographic data where the same top four features had the highest predictive power for both MBI-Q5 and MBI. We elaborate on the significance of the factors with the highest predictive power for both target variables in the "Discussion" section.

### Descriptive Statistics

The features listed in the "Feature predictions" section were translated into the factors that had the highest influence on MBI_Q5 and MBI by turning the survey questions associated with each feature into a factor. As a final step in evaluating the results of the respective ML models to predict MBI_Q5 and MBI, the full survey dataset was analysed according to the factors with the highest influence to predict the two target variables (MBI_Q5 and MBI).

**Fig. 7:**
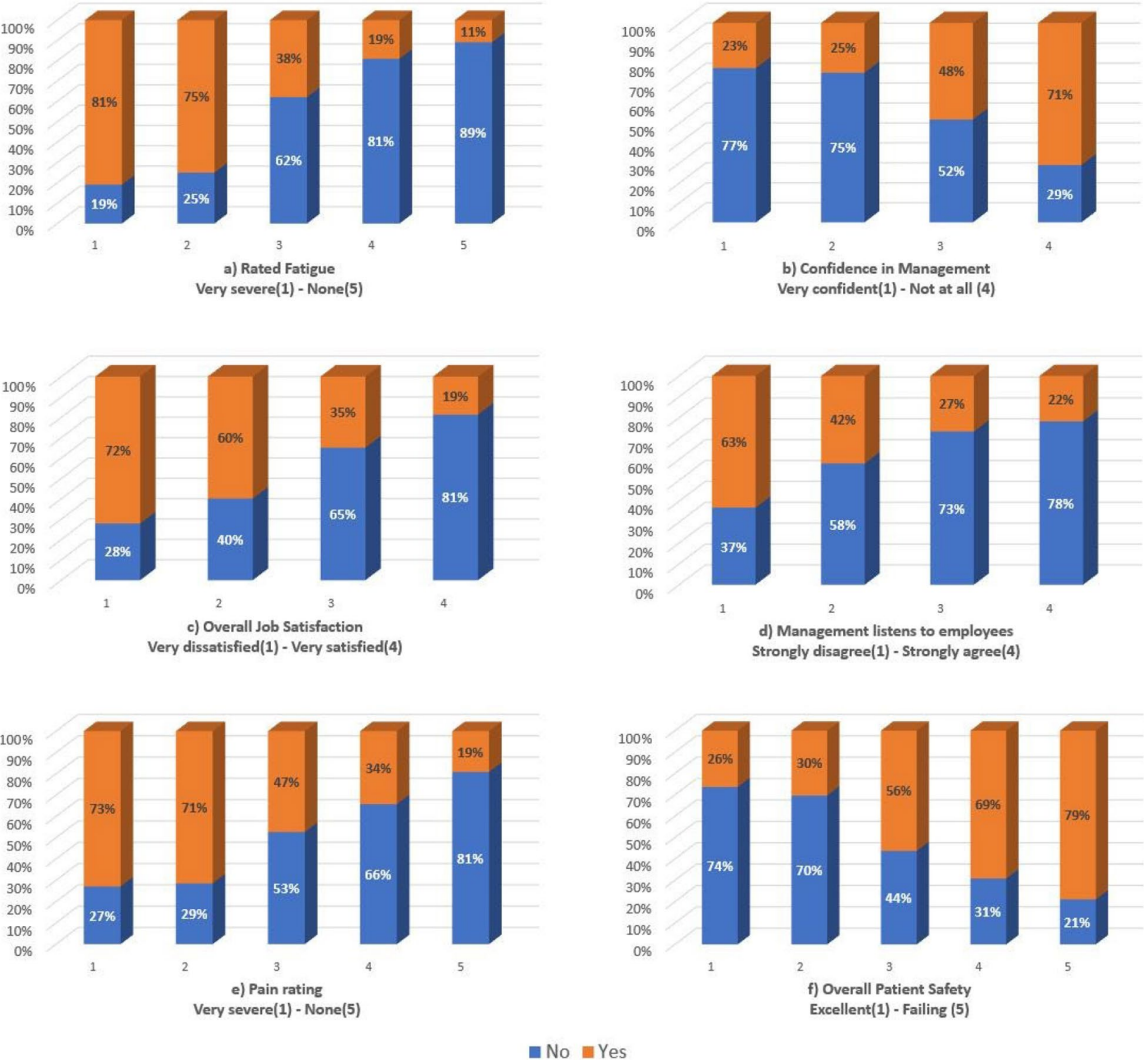
Analysis of the features with the highest predicting power on MBI_Q5

Figure 7 depicts the analysis of the top six factors that had the highest predictive power to predict that nurses will indicate they feel burned out from their work once a week or more (MBI_Q5).

This analysis corresponds with the output of the ML model on MBI_Q5 in that of all respondents who rated their own fatigue as very severe, 81% indicated that they feel burned out from their work once a week or more. Of the respondents who had no confidence in management, an overwhelming (71%) were positive MBI_Q5 cases. Participants who had a very severe pain rating also related 73% of positive self-reported burnout cases. Nurses who indicated that they felt burned out from their work once a week or more were also 63% of the respondents who strongly disagreed that management listens to employees.

**Fig. 8:**
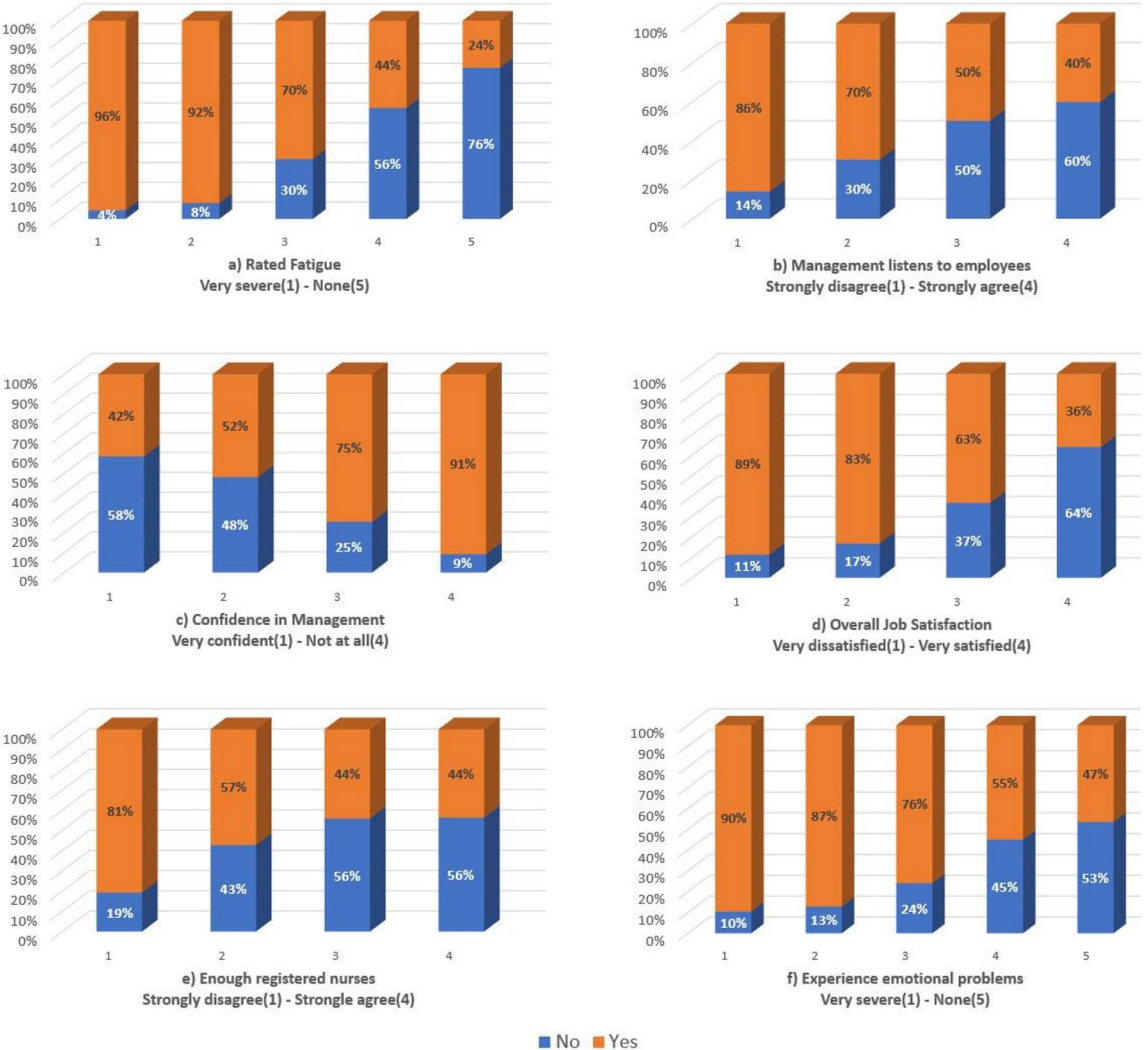
Analysis of the features with the highest predicting power on MBI

Similarly, the response of the factors that most prominently predict high emotional exhaustion is provided in Fig. 8.

The findings show that 96% of all nurses who rate their fatigue as very severe also suffered from high emotional exhaustion. Additionally, 86% of nurses who strongly disagree that management listens to employee problems suffer from high emotional exhaustion. This corresponds to 91% of nurses who have no confidence in management and suffer from high emotional exhaustion. 81% of nurses who strongly disagreed that there are enough registered nurses to provide care also showed high levels of emotional exhaustion. With 90% of respondents who experience severe emotional problems also experiencing high emotional exhaustion, the factors extracted from the ML model to predict high emotional exhaustion can be concluded as plausible.

## Discussion

To address the research objective of this research, we developed an ML model from full survey data to determine the factors that most prominently predict that nurses in South Africa will respond in the 9-item Maslach Burnout Inventory tool that they feel burned out from their work once a week or more (MBI_Q5). The same approach was followed to address the second research objective, where an ML model was developed that can identify the factors that most strongly predict that nurses in South Africa will score 27 or more in the 9-item Maslach Burnout inventory tool and therefore experience high emotional exhaustion (MBI). The third research objective was addressed by comparing the predictions of the ML models on full survey data versus the predictions on demographic data only.

The predictions of the ML models indicated an accuracy score of 75.8% for the MBI_Q5 with Subset 1 compared to 60.4% when only demographic data were included. Likewise, for the MBI indicator, Subset 2 gave an accuracy score of 76.8% compared to 68.5% for the demographic subset. It is, therefore, clear that the inclusion of the survey data had a significant impact on improving the accuracy of both self-reporting feelings of burnout and emotional exhaustion. When comparing the features listed as most significant for predicting the target variables, for the ML models with and without the full survey data, none of the demographic features like absenteeism, number of years worked, age and specialism were listed under the top features of the more accurate model.

### Full survey features

Focusing on factors with high predictive power from the full survey datasets, the fatigue of nurses was rated first in the prediction of both MBI_Q5 and MBI in this study. Overall fatigue has been associated with burnout in several studies [58], also during the pandemic [59]. Nurses are prone to experiencing fatigue due to high-stress situations and work context factors such as a high workload [60]. Bayes et al. [61] explain that experiencing fatigue is both a physical contributor and a physiological result of burnout. Thus, the predictive nature of fatigue in the development of burnout is already established.

However, fatigue is not the only internally focused predictor of burnout. The predicting factors of nurses indicating that they frequently experienced pain or emotional symptoms were also listed as factors with high predictive power in the ML models. Experiencing a high pain rating was the fifth predictor for MBI_Q5, while experiencing emotional problems was the sixth predictor for high emotional exhaustion (MBI). While the association between burnout and frequently experiencing pain, and likewise burnout and emotional symptoms are established [21, 62], these symptoms have not previously been seen as predictors of burnout or emotional exhaustion, although systematic reviews do highlight them as occupational consequences of burnout [63].

There are also job-related factors predicting that nurses will indicate that they feel burned out from their work once a week or more frequently and suffer from emotional exhaustion. Overall job satisfaction in the survey was comprised of the satisfaction rating in relation to: work schedule; opportunities for advancement; independence at work; professional status; salary or wages; educational opportunities; appreciation, recognition and rewards; immediate supervisor or manager; co-workers; and work-life balance. These elements of job satisfaction could all be included as the organisations’ responsibilities. While Omer et al. [64] and Hodkinson et al. [65] identify burnout as a predictor of job satisfaction, Antonio et al. [66] acknowledge that job satisfaction can be a positive mediator in mitigating burnout. The predictive value of job satisfaction in the development of burnout is thus supported.

Two items representing organisation-related factors, namely “management that listens and responds to employee concerns” and “how confident are you that management will act to resolve problems in patient care that nurses identify?” emphasise the predictive value of good management when it comes to the employee indicating that they feel burned out from their work once a week or more (MBI_Q5) and high emotional exhaustion incidence (MBI). The item on nursing staff perspective on whether management listens to employee concerns rated fourth in factors impacting nurse MBI_Q5 and second in factors predicting high emotional exhaustion, while the item on confidence in management rated second in predicting MBI_Q5 and third in predicting MBI. McCleery [67] agrees with the correlation between managerial support and nurses’ burnout, while Peng and Lihua [68] explain that managers’ actions can directly affect nurse burnout, while also indirectly affecting burnout through work stress. Thus, agreement on the predictive nature of managerial support in the development of burnout exists.

Healthcare system factors include resource adequacy. Specifically, the sufficiency of registered nurses was identified as the fifth major predictor of the development of emotional exhaustion in nurses. The impact of insufficient staffing on burnout and emotional exhaustion is well-established in the literature [69–71].

Focusing externally, perceived patient safety was seen as the sixth major predictor for nurse burnout. Although the consensus is that patient safety is impacted by nurse burnout [72–74], there is also evidence that poor quality of care and patient safety outcomes can result in burnout [75].

Thus, in mitigating emotional exhaustion and feelings of burnout amongst nurses, strategies to limit nurse fatigue, support physical health, enhance job satisfaction, build trust in management, and address staffing concerns should be prioritised. On the nursing practice level, this might include fostering a climate of appreciation and recognition from nursing colleagues [2] and managers [76] to enhance job satisfaction and creating wellness-related programs for nurses [77] that might mitigate nurse fatigue and physical health symptoms such as pain. Wang et al. [76] emphasise the onus on managers in positive nurse and patient outcomes, relating to the need for trust in managers. Also on management level, nurse staffing shortages should be addressed as priority, as Aiken et al. [2] agree with our predictive findings in that nurse staffing is deemed more effective than any other intervention in championing nurses’ personal wellbeing. Nursing education should include stress management training [77], while overall training of sufficient numbers of nurses would further alleviate the staff shortages [78]. Contextual research on retention strategies [78] might address fatigue due to work overload, poor nurse job satisfaction and staffing shortages [2].

### Demographic features

The evidence on the impact of demographic characteristics on feelings of burnout and emotional exhaustion, is conflicting. While social-demographic variables are included in most studies on burnout, some systematic reviews contend that their influence on burnout is negligible or non-existent [79]. Other systematic reviews assert that demographics do have a definitive effect on the risk of burnout [80, 81]. The results of this study provide empirical evidence, that demographic factors had less impact on burnout risk, than other personal and job-related factors, but highlighted the following five demographic factors as having the most impact, namely absenteeism, work experience, age, the speciality of the unit the nurse is working at, and the healthcare sector employed in.

Lee et al. [82] declared the association between burnout, and especially, emotional exhaustion and absenteeism, emphasizing that absenteeism is caused by higher levels of burnout. On the contrary, Sabzi et al. [83] argue that the converse of this relationship might also be true and present evidence that nurses might experience their own absenteeism as indicators of developing burnout (highest rated demographics factor) and specifically emotional exhaustion (fourth-rated demographics factor).

Another two demographic factors, years of work experience within a specific hospital and years of experience as a nurse, might also impact the development of burnout or emotional exhaustion. Liao et al. [84] found a significant association between the number of years of experience and emotional exhaustion albeit non-linear, while Wang et al. [85] explained that the gap between job expectations and professional nurses (or low fit within a post) might lead to increased levels of burnout amongst nurses with less experience. Wang et al. [85] elaborate that nurses who had worked for three to ten years were prone to emotional exhaustion. In this study, years worked in a specific hospital ranked second and first in predicting that nurses will indicate they have feelings of burnout from their work once a week or more and high emotional exhaustion, respectively, while years worked as a nurse ranked third as a predictor for both burnout and emotional exhaustion.

Related to experience, Moya-Salazar et al. [86] found that younger nurses (specifically aged 20–30 years) presented with the highest levels of burnout, although no explanation for this outcome was offered. Beier et al. [87] provide more insight into this matter by not only presenting the relationship between younger age and increased burnout but also the positive association between age and better-developed positive coping strategies. Age, in the study, was rated as the fourth and second demographic factor impacting the prediction of MBI_Q5 and MBI, respectively.

Adding to demographic factors, the notion that the speciality of nurses impacts their perception of burnout is not novel. According to Ashipala and Ngole [88], significant differences in burnout incidence amongst nurses have been noted between specialties. This could be due to the difference in workload, such as workarounds required during medication administration [21], or speciality specific factors, such as being faced with higher patient mortality rates [89]. In this study, the nurse speciality rated fifth as the demographic factor in predicting MBI_Q5 and MBI.

Contrary to Coetzee et al. [90], the ML models did not identify the sector of healthcare (private versus public), as a feature that contributes significantly towards predicting either of the two burnout variables; in fact, this feature’s predictive power is negligible for both full survey and demographic data models in burnout self-reporting. It had the sixth-highest predictive power for emotional exhaustion on demographic data. Still, with a predictive power of 5.0%, it has less predictive power than any other feature on average, given that there are 11 features, as discussed in the"Feature predictions" section. Its predictive power is also negligible in predicting emotional exhaustion on full survey data.

A limitation of our study is that the data collection period coincided with different levels of severity of the COVID-19 pandemic. One may, therefore, argue that the factors associated with self-reported burnout and emotional exhaustion are only valid under pandemic conditions. However, we contend that the COVID-19 pandemic exacerbated risk factors associated with burnout and emotional exhaustion amongst nurses in South Africa. The data collected during these strenuous circumstances is therefore useful to model predictions of feelings of burnout and emotional exhaustion on. Furthermore, classification models were used for predicting risk factors associated with positive self-reported feelings of burnout and emotional exhaustion. Future research could use regression models to predict self-reported burnout and emotional exhaustion frequency.

## Conclusion

In this research, we set out to identify the distinct factors that can strongly predict feelings of burnout and emotional exhaustion among nurses in South Africa. This was achieved by developing supervised ML models with high accuracy capable of predicting cases where feelings of burnout occurred at least once a week, as well as emotional exhaustion, from which these predictive factors were extracted.

The first two objectives of this research were to use ML models to determine the factors that most strongly predict that nurses will indicate that they feel burned out from their work (MBI_Q5) or suffer from high emotional exhaustion (MBI). The third objective was to compare the accuracy of these predictions by using full survey data compared to demographic data only. The two ML models investigated to predict MBI_Q5 and MBI both returned high accuracy scores of 75.8% and 76.8% respectively. This means that the predictive power of the full survey data is significant with higher accuracy values than previous ML models reported by [19].

None of the demographic variables were note-worthy predictors of burnout or emotional exhaustion when compared to the organizational factors influencing nurse and patient outcomes. While the authors acknowledge that personal demographics might impact the development of burnout in nurses, the predictive models presented here suggest that factors such as fatigue, confidence in management, job satisfaction, managerial support, physical and emotional symptoms, patients’ safety, and human resource adequacy ultimately outweighs demographic considerations in determining factors leading to nurse burnout. Interventions on burnout are either individual-focused or organisational-focused, or a combination of the two [91]. The impact of individual-focused interventions is limited [92]. It often lasts for a short time frame, with the most impact being seen in the creation of a positive work environment [93, 94], which was highlighted in a meta-analysis as having the most impact on both nurse outcomes (burnout) and patient outcomes [95]. This calls for a perspective shift from individual nurses towards systems and work environments that will protect nurses in South Africa.

## Data Availability

Due to the nature of the research, due to [ethical/legal/commercial] supporting data is not available.

## Abbreviations

AUC: Area under the curve
FN: False Negative
FP: False Positive
HEE: High Emotional Exhaustion
MBI: Maslach Burnout Inventory
ML: Machine Learning
RF: Random Forest
RGS: Random grid search
ROC: Receiver operating characteristic
SBO: Self-Reported Burnout
STROBE: Strengthening the Reporting of Observational Studies in Epidemiology
TP: True Positive
TN: True Negative
